# Maintenance of plasma glucose variability after an acute session of aerobic exercise despite changes in insulin and glucagon-like peptide-1 levels in type 2 diabetes

**DOI:** 10.20945/2359-3997000000482

**Published:** 2022-05-25

**Authors:** Patrícia Martins Bock, Raíssa Borges Monteiro, Gabriela Berlanda, Karina Rabello Casali, Beatriz D. Schaan

**Affiliations:** 1 Hospital de Clínicas de Porto Alegre Laboratório de Pesquisa em Fisiopatologia do Exercício Porto Alegre RS Brasil Laboratório de Pesquisa em Fisiopatologia do Exercício, Hospital de Clínicas de Porto Alegre, Porto Alegre, RS, Brasil; 2 Faculdades Integradas de Taquara Taquara RS Brasil Faculdades Integradas de Taquara, Taquara, RS, Brasil; 3 Universidade Federal do Rio Grande do Sul Faculdade de Medicina Departamento de Clínica Médica Porto Alegre RS Brasil Universidade Federal do Rio Grande do Sul, Faculdade de Medicina, Departamento de Clínica Médica, Programa de Pós-graduação em Ciências Médicas – Endocrinologia, Porto Alegre, RS, Brasil; 4 Hospital de Clínicas de Porto Alegre Porto Alegre RS Brasil Hospital de Clínicas de Porto Alegre, Porto Alegre, RS, Brasil; 5 Universidade Federal de São Paulo Departamento de Ciência e Tecnologia São José dos Campos SP Brasil Universidade Federal de São Paulo, Departamento de Ciência e Tecnologia, São José dos Campos, SP, Brasil; 6 Universidade Federal do Rio Grande do Sul Programa de Pós-graduação em Cardiologia Porto Alegre RS Brasil Programa de Pós-graduação em Cardiologia, Universidade Federal do Rio Grande do Sul, Porto Alegre, RS, Brasil

**Keywords:** Blood glucose, diabetes mellitus, glycemic variability, hormones

## Abstract

**Objective:**

The present study aimed to evaluate glucose variability and hormonal responses during and after an aerobic exercise session performed after breakfast in type 2 diabetes patients treated with metformin.

**Materials and methods:**

In this quasi-experimental study individuals underwent clinical and laboratory evaluations and maximal exercise test. After two weeks an aerobic exercise session (30 minutes at 60%-70% of the peak heart rate) was performed. At rest, during and after the exercise session, glucose variability (mean amplitude glucose excursions, glucose coefficient of variation, and glucose standard deviation) and levels of plasma glucose, insulin, glucagon, and glucagon-like-peptide-1 were evaluated.

**Results:**

Thirteen patients were enrolled in the study. Plasma glucose increased at 15 minutes during the exercise session (244.6 ± 61.9 mg/dL), and decreased at 60 min after exercise (195.6 ± 50.0 mg/dL). Glucose variability did not show any difference before and after exercise. Insulin levels at 15 min [27.1 µU/mL (14.2-42.1)] and 30 min [26.3 µU/mL (14.6-37.4)] during the exercise were higher than those at fasting [11.2 µU/mL (6.7-14.9)] but decreased 60 minutes after exercise (90 minutes) [16.6 µU/mL (8.7-31.7)]. Glucagon levels did not show any difference. GLP-1 levels increased at 30 min [7.9 pmol/L (7.1-9.2)] during exercise and decreased 60 min after exercise (90 minutes) [7.7 pmol/L (6.8-8.5)].

**Conclusion:**

Subjects with type 2 diabetes presented expected changes in insulin, glucagon and GLP-1 levels after breakfast and a single aerobic exercise session, not accompanied by glycemic variability changes.

## INTRODUCTION

Type 2 diabetes mellitus is characterized by relative insulin deficiency and insulin resistance. Glucose control is primarily assessed based on the level of glycated hemoglobin (HbA1c), and one-point reduction in the HbA1c level may reduce the risk of cardiovascular disease outcomes by 10% over a period of several years (1). Assessing short-term glucose variability by continuous glucose monitoring (CGM) may add to the evaluation of glucose control because glucose fluctuations, which may lead to hypoglycemia or postprandial hyperglycemia, can be noted on-time and managed properly (2). Wide variability in glucose levels leads to the production of reactive oxygen species and causes endothelial dysfunction, possibly leading to an increased risk of cardiovascular disease in diabetes (3).

Individuals with type 2 diabetes should ideally perform both aerobic and resistance exercise training to attain optimal glycemic control. Performing structured exercise for at least 150 min/week can cause a decline of 0.67% in the HbA1c level (4). Moreover, exercise performed in the morning and after breakfast can lead to a better glucose-lowering effect throughout the day and a lower risk of nocturnal hypoglycemia (5). Glucose variability after acute aerobic and eccentric exercise was reported to be decreased in healthy subjects (6), as well as in patients with diabetes after acute aerobic and combined aerobic and resistance exercise (7). However, a three-month treatment with vildagliptin or glibenclamide added to metformin did not lead to any change in glucose variability after acute aerobic exercise in individuals with type 2 diabetes (8). A comparison of the effects of moderate exercise performed in either the fasting or postprandial state on glucose variability showed that performing moderate exercise in the postprandial state after breakfast tended to decrease glucose excursions compared to the exercise performed in the fasting state in a small sample of pre-diabetes individuals (9). It is worth noting that none of the aforementioned studies evaluated at once glucose variability and hormonal changes after acute aerobic exercise, in postprandial state, in individuals with type 2 diabetes treated only with metformin.

Along with changes in glucose variability, exercise can elicit changes in multiple hormones, including insulin, glucagon, and glucagon-like peptide-1 (GLP-1) (10). In healthy adults, acute aerobic exercise elicits lower insulin and higher glucagon levels, with maintenance of plasma in the fasted state; in the postprandial state, however, decreased glucose and insulin concentrations may be seen (11). The incretin GLP-1 plays a crucial role in maintaining metabolic homeostasis, since it induces postprandial insulin release and suppresses glucagon secretion; although there are no systematic differences in the nutrient-induced secretion of GLP-1 between healthy and type 2 diabetic subjects, there are characteristic abnormalities in the insulinotropic and glucagonostatic activities among them (12). The hormonal response to exercise could be affected in diabetes: postprandial suppression of glucagon, increase of GLP-1, and no change in insulin levels was observed in diabetes patients one day after performing two long bouts of exercise (13). Although other studies have shown no change in insulin levels after acute sessions of aerobic exercise (14,15), other authors reported a reduction in these levels (16), while glucagon levels were found to have increased after aerobic exercise (17), and GLP-1 levels decreased during post-dinner resistance exercise (18). Thus, the present study aimed to evaluate glucose variability and hormonal responses during and after an aerobic exercise session performed after breakfast in type 2 diabetes subjects, treated with metformin.

## MATERIALS AND METHODS

### Research design and participants

This was a quasi-experimental study (nonrandomized, pre-post intervention). Patients were recruited from the endocrinology outpatient clinic at a tertiary teaching hospital and through a website. Inclusion criteria were age older than 18 years, presence of type 2 diabetes mellitus, use of metformin, recent HbA1c level between 7.5% and 10%, and no involvement in regular physical activity (>1x/week). Exclusion criteria were current smoking, body mass index (BMI) > 40 kg/m^2^, proliferative diabetic retinopathy, ischemic heart disease, peripheral vascular disease, cognitive decline or dementia, recent neurological event, severe depression, current diagnosed cancer, lactose intolerance, hepatic enzyme levels threefold higher than the reference values, glomerular filtration rate lower than 60 mL/min, blood pressure (BP) > 180/100 mmHg at rest, use of insulin or other agents except metformin, and untreated thyroid disease.

This study was conducted in accordance with the Declaration of Helsinki and was approved by the Scientific Committee and Research Ethical Commission of *Hospital de Clínicas de Porto Alegre* (Brazil) (Certificate of Presentation for Ethical Appreciation #10662912300005327).

### Data collection

Eligible individuals underwent clinical and laboratory evaluations, including clinical characteristics, blood pressure measurement, and fasting blood sample collection for HbA1c. After this evaluation, patients performed a symptom-limited maximal exercise test on a cycle ergometer to determine peak oxygen consumption (VO_2_peak) and peak heart rate. The test was carried out in increments of 15 to 20 Watts *per* minute, maintaining a frequency of 60 rpm or more until exhaustion was reached. Oxygen consumption (VO_2_) and carbon dioxide production (VCO_2_) were determined by a breath-by-breath computerized gas exchange system and analyzed using 20-second averaging system (Oxycon Delta; VIASYS Healthcare GmbH, Jaeger, Germany). Peak VO_2_and peak heart rate were defined as the greatest values reached in the last 30 seconds of exercise testing (19).

Two weeks after clinical and laboratory evaluations and maximal exercise test, an aerobic exercise session starting with a 5-min warm-up followed by 30 minutes at 60%-70% of the peak heart rate, (as determined in the maximal exercise test) was performed, with 5 minutes of cooling after exercise. Glucose variability (mean amplitude glucose excursions, glucose coefficient of variation, and glucose standard deviation), and metabolic and hormone variables (glucose, insulin, glucagon, glucagon-like-peptide-1) were measured before, during, and after the exercise session.

The study protocol was conducted during three days, according to the following procedure:

- Day 1: Subjects were admitted to the laboratory at approximately 08:00 AM, 24 hours before the exercise session, when the glucose sensor (i-Pro2 digital recorder; Medtronic Mini-Med Inc., Northridge, CA, USA) was inserted subcutaneously.
- Day 2: Subjects were admitted to the laboratory at approximately 08:00 AM. The submaximal exercise session was performed after intake of a standard breakfast (500 kcal; 60% complex carbohydrate, 30% fat, and 10% protein). Blood samples were collected 60 min before (fasting), immediately before (after breakfast, 0 min), 15 and 30 minutes during the exercise session, and 60 minutes after exercise. Blood samples were obtained to measure the levels of plasma glucose, insulin, glucagon, and GLP-1. After exercise, subjects were allowed to eat ad-libitum.
- Day 3: Subjects were admitted to the laboratory at approximately 08:00 AM, and the glucose sensor was removed.

### Measurements

The continuous glucose monitoring system (CGMS), which measured glucose levels every 5 min, was used to obtain a series of glucose values. Glucose variability was evaluated based on the following indices, using conventional analysis: mean amplitude glucose excursions (MAGE), glucose coefficient of variation (CV%), and glucose standard deviation (SD). These indices, except MAGE, were calculated in a 6-hour timeframe of glucose values to obtain the measures according to the specific period of the day. The MAGE index was calculated for the whole signal, and its calculation is based on the differences between peaks and nadir points, considering those points that are higher than 1SD (20).

Venous blood samples were drawn from the antecubital vein in gel-clot Vacutainer™ tubes for measuring HbA1c, plasma glucose, and insulin levels and ethylene diamine tetra-acetic acid (EDTA)-coated tubes for measuring glucagon and GLP-1 levels. Subsequently, 2.0 mL of blood was transferred into a tube containing a dipeptidyl peptidase (DPP-4) inhibitor to prevent the degradation of active GLP-1. The blood collected in the EDTA tubes was centrifuged for 10 min at 1,000 *g* to separate plasma, and then the plasma was stored at -80 °C for further analysis.

HbA1c was analyzed by ion-exchange high-performance liquid chromatography (Merck-Hitachi L-9100 HbA1c analyzer; Merck, Darmstadt, Germany). Plasma glucose levels were analyzed according to the glucose oxidase method (Sigma-Aldrich, St Louis, MO, USA), insulin levels were analyzed according to chemiluminescent enzyme immunoassay (Immulite 1000 Analyzer; Siemens Healthcare Diagnostics, Deerfield, IL, USA), glucagon levels were analyzed according to colorimetric enzyme immunoassay (R&D Systems, Minneapolis, MN, USA), and GLP-1 levels were analyzed according to fluorescence enzyme immunoassay (EMD Millipore, Billerica, MA, USA). The correction of plasma glucose and hormone levels for exercise-induced changes in plasma volume was not made because the aerobic exercise session lasted 30 minutes at 60%-70% of the peak heart rate, when no plasma volume changes were expected (21).

### Statistical analyses

Data were analyzed using SPSS software (Statistical Package for the Social Sciences; version 18.0 for Windows, SPSS Inc., Chicago, USA). Values are expressed as mean ± SE for parametric variables and median (P25-P75) for non-parametric variables. Categorical variables are expressed as number (%). Comparisons were tested using paired-samples t-test, Wilcoxon signed-rank test, or Friedman’s test (non-parametric repeated measures ANOVA), followed by Dunn’s post-hoc test (p < 0.05).

## RESULTS

**Figure 1:**
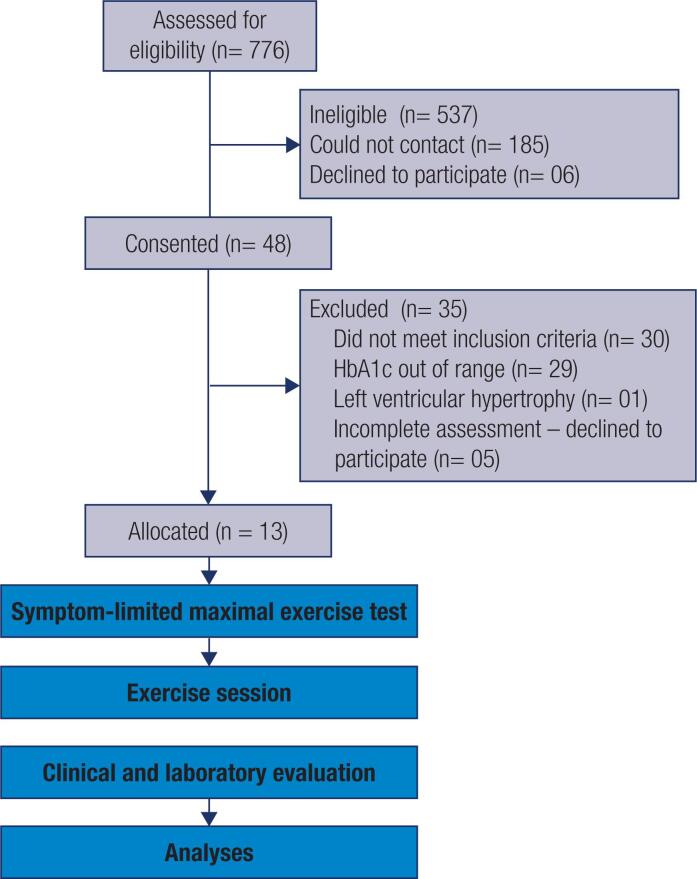
Flow diagram.

Thirteen individuals with diabetes were included in the study. The Figure 1 shows the flowchart of the participant selection process through each stage.

Table 1 shows patients’ characteristics. They were 56.2 ± 1.5 years old, predominantly men; 83% were overweight/obese and had diabetes for 3.5 years (1.8-12 years). Most patients had moderately reduced (25.7 ± 1.9) VO_2_ peak values.

**Tabla 1:** Characteristics of the studied patients (n = 13)

Age (years)		56.2 ± 1.5
Male (n)		8
Diabetes duration (years)		3.5 (1.8-12)
BMI (kg/m²)		29.6 ± 1.2
SBP (mm/Hg)		133.5 ± 3.3
DBP (mm/Hg)		82.6 ± 2.2
VO2 peak (mL/kg/min)		25.7 ± 1.9
HbA1c (%)		8.8 ± 0.3
Medications		
	ACEi (n)	1
	ß-blockers (n)	3
	ARB (n)	4
	Calcium-channel blockers (n)	4
	Daily dose of metformin (mg)	1,700 (850-2,550)

HbA1c: glycated hemoglobin; VO_2_peak: peak oxygen uptake per kilogram of body weight; BMI: body mass index; SBP: systolic blood pressure; DBP: diastolic blood pressure; ACEi: angiotensin converting enzyme inhibitor; ARB: angiotensin II receptor blocker. Continuous variables are expressed as mean ± SE or median [interquartile range (p25-p75)]. Categorical variables are expressed as number.

**Figure 2:**
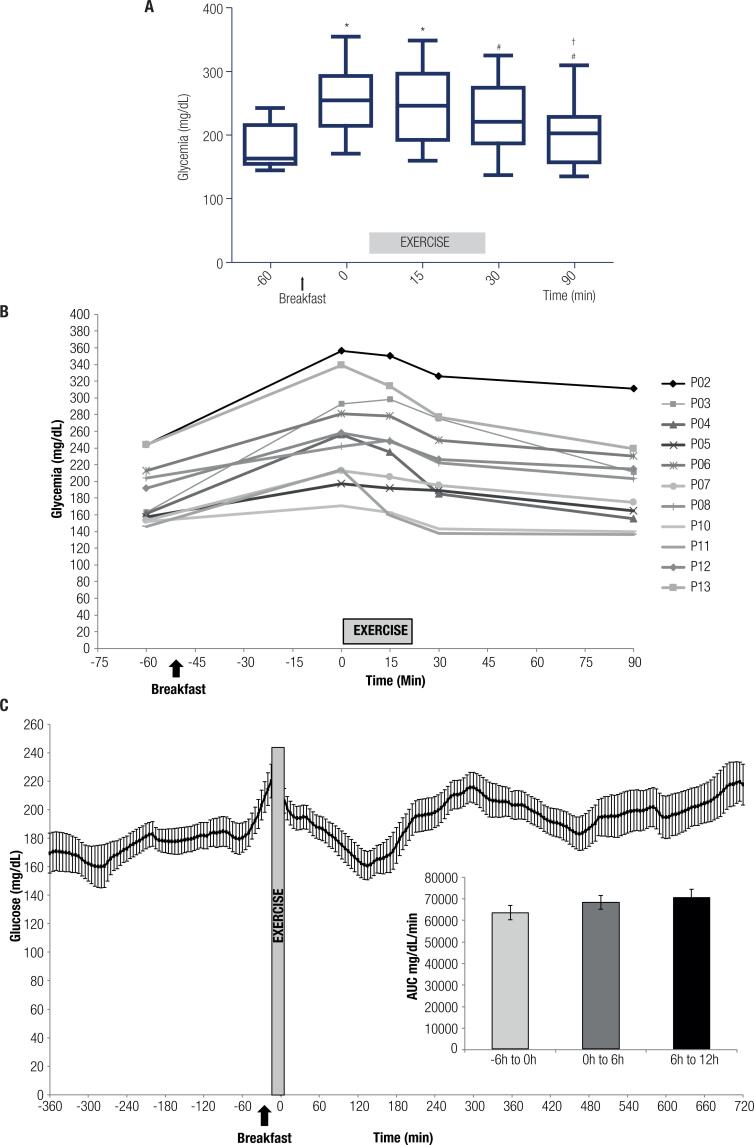
Plasma glucose at baseline (fasting, -60’), before (0’), during (15’), and after exercise (30’ and 90’ after the beginning and 60’ of recovery) (panel a). Individual data of plasma glucose at baseline (fasting, -60’), before (0’), during (15’), and after exercise (30’ and 90’ after the beginning and 60’ of recovery) (panel b). Glucose (continuous glucose monitoring, sampled every 5 minutes) before and after exercise (panel c). Incremental area under the curves (AUC) are shown at the inset (panel c). Data are presented as mean values and standard error. For panel A: *p < 0.05 vs. -60’; # p < 0.05 vs. 0’; † p < 0.05 vs. 15’. n = 11.

Figure 2 (panel a) shows plasma glucose levels 60 min before exercise (fasting, 185.2± 35.3mg/dL), immediately before exercise (after breakfast – 0 min, 256.4± 57.8 mg/dL), 15 (244.6± 61.9 mg/dL) and 30 minutes during the exercise session (220.5± 58.2 mg/dL), and 90 minutes after the beginning of the exercise session (60 minutes after exercise, 195.6± 50.0 mg/dL). Plasma glucose levels increased after breakfast and at 15 minutes during the exercise session, and decreased at 30 min and 60 min after exercise (p < 0.05). Panel b shows individual data of plasma glucose. Panel c shows glucose values obtained from the CGMS 60 minutes before exercise (fasting) and during the first 12 hours after exercise. Considering the incremental area under the curve (AUC – panel c, insets), no differences were found.

Glucose variability, evaluated in a time frame of 6 hours by CV and SD, did not show any difference before and after the exercise (Table 2). Before the exercise, MAGE (in a time frame of 24 hours) was 61.6 (40.8-103.3) mg/dL, and after exercise, it was 65.8 (60.2-100.2) mg/dL (p = 0.646).

**Tabla 2:** Glucose variability evaluated before and after the exercise session (n = 12)

		Before exercise	After exercise	
		-6h to 0h	0h to 6h	6h to 12h
Glucose (mg/dL)	Mean ± SE	179.3 ± 9.2	195.4 ± 8.9	198.7 ± 11.3
	Median (p25-p75)	187.6 (155.8 – 204.3)	196.2 (170.2 – 220.2)	201.0 (172.9 – 232.9)
CV (%)	Mean ± SE	11.6 ± 1.5	11.4 ± 1.7	11.9 ± 1.6
	Median (p25-p75)	10. 2 (9.7 – 15.0)	10.4 (5.8 – 17.4)	11.6 (8.7 – 17.3)
Glucose SD (mg/dL)	Mean ± SE	21.8 ± 1.8	22.0 ± 3.5	22.5 ± 2.5
	Median (p25-p75)	20.8 (16.7 – 29.5)	22.6 (10.2 – 31.1)	21.3 (15.4 – 31.4)

Glucose variability evaluated in patients with type 2 diabetes inadequately controlled with metformin therapy, before and after aerobic exercise. CV%: coefficient of variation; SE: standard error, SD: standard deviation. Data are expressed as mean ± SE and median [interquartile range (p25-p75)]. Comparisons were tested by Friedman’s test. No differences were found.

Plasma insulin, glucagon, and GLP-1 levels were analyzed 60 minutes before the exercise (fasting), immediately before the exercise (after breakfast, 0 minute), 15 and 30 minutes during the exercise session, and 90 minutes after the beginning of the exercise session (60 minutes after exercise) (Table 3). Insulin levels after breakfast [24.2 µU/mL (16.6-34.6)] and at 15 minutes [27.1 µU/mL (14.2-42.1)]and 30 minutes (exercise) [26.3 µU/mL (14.6-37.4)] were higher than those at fasting [11.2 µU/mL (6.7-14.9)] but decreased 60 minutes after exercise (90 minutes) [16.6 µU/mL (8.7-31.7)]. The intra-assay coefficient of variation for insulinemia was 2.7% and the inter-assay (n = 3) was 3.4%. Glucagon levels did not show any difference in any of the studied moments. The intra-assay coefficient of variation for glucagon levels was 4.3% and the inter-assay was 8.3%. GLP-1 was increased after breakfast [8.3 pmol/L (6.8-9.1)] and at 30 minutes (exercise) [7.9 pmol/L (7.1-9.2)] and decreased 60 minutes after exercise (90 min) [7.7 pmol/L (6.8-8.5)]. The intra-assay coefficient of variation for GLP-1 levels was 3.6% and the inter-assay was 7.2%.

**Tabla 3:** Hormonal levels before and after the exercise session (n = 13)

Insulin (µU/mL)		
	Fasting	11.2 (6.7 – 14.9)
	0 min	24.2 (16.6 – 34.6)*
	15 min	27.1 (14.2 – 42.1)*
	30 min	26.3 (14.6 – 37.4)*
	90 min	16.6 (8.7 – 31.7)
GLP-1 (pmol/L)		
	Fasting	7.0 (6.4 – 7.7)
	0 min	8.3 (6.8 – 9.1)*
	15 min	7.7 (7.0 – 9.1)
	30 min	7.9 (7.1 – 9.2)*
	90 min	7.7 (6.8 – 8.5)
Glucagon (pg/mL)		
	Fasting	117.3 (113.1 – 135.9)
	0 min	128.1 (115.3 – 142.6)
	15 min	119.6 (113.6 – 139.1)
	30 min	131.9 (116.9 – 145.6)
	90 min	119.6 (116.5 – 141.8)

Hormonal levels at fasting (-60 min), immediately before (0 min), during (15 min and 30 min), and 90 min after the beginning of the exercise (60 min of recovery) in patients with type 2 diabetes inadequately controlled with metformin therapy. GLP-1: glucagon like peptide-1. Variables are expressed as median [interquartile range (p25-p75)]. Comparisons were tested by Friedman’s test.

* p<0.05 *vs*. 0 min.

## DISCUSSION

In this study, we showed that plasma glucose levels increased after breakfast and decreased in the recovery period after acute aerobic exercise session until 90 min, in type 2 diabetes subjects. Interestingly, glucose variability did not show any difference either before or after the breakfast and the acute bout of exercise. These changes occurred along with the rise of insulin and GLP-1 levels and no modification in glucagon levels.

Despite its clinical significance, there is no consensus on the optimum method for characterizing glucose variability. Over the years, various metrics quantifying glucose variability have been introduced, and it is clear that no index is better than the other, but the indices are probably complementary (22). Glucose variability analyzed by CV% was approximately 10%, before and after the exercise, in accordance with a previous study conducted by our group (8). Usually, a cutoff value of 36% is considered as labile glucose control in subjects with either type 1 or type 2 diabetes (23). This finding is in accordance with that of a previous study; however, with a very different intervention – just interrupting prolonged sitting (24) – which is not comparable to the effort, the subjects were submitted in the present study. Another study showed lowered CV% after moderate-intensive exercise, but the evaluation was performed in a longer time frame (12 h), as compared to the exercise we proposed in this study (25).

The other index we used to evaluate glucose variability, glucose SD, was also unchanged by the acute bout of aerobic exercise, which is different from the findings reported in the study of Li and cols. with a 12-h observation period, as reported for CV% (25), but similar to that observed by Dempsey and cols. Although differences in the intervention are clear, these authors discuss that the effects they observed in some glucose variability indices were not apparent following statistical adjustment for mean glucose levels. These findings, together with the lack of CV% difference (which directly normalizes for mean glucose), point to a similar relative magnitude of glucose fluctuations around lower mean values for the sedentary and non-sedentary conditions, rather than less variability *per se* (24). However, in agreement with our results, a single bout of moderate-intensity cycling exercise performed in a large group of 60 patients with type 2 diabetes under strict dietary standardization showed no significant changes in SD, in both non-insulin- and insulin-treated patients (26). Moreover, systematic reviews published on this topic reveal a high clinical and statistical heterogeneity, also preventing the reader from being fully confident in their conclusions (27,28).

The third index used to evaluate glucose variability was MAGE, and we found no change after the acute bout of aerobic exercise performed after breakfast. In accordance, Terada and cols. observed that a bout of high-intensity interval exercise or energy-matched moderate-intensity continuous exercise performed in the fasting state reduces MAGE. The same result was not observed in the post-breakfast state, suggesting that exogenous nutrient availability during exercise hampers favorable changes in glucose levels that persist hours after exercise (29). Furthermore, MAGE was neither reduced after 30 min of continuous exercise or high-intensity interval training in men with type 2 diabetes under standardized dietary intake (30), nor after one session of 20-minute moderate-intensity treadmill walking after dinner (25).

The effect of the acute aerobic exercise session performed in the postprandial state on glucose levels observed here is the classical well-known effect of glucose reduction, which is related to increased plasma membrane content of GLUT4 (31). Overall, lower glucose levels were described at the end of the exercise performed in the fasting state or after lunch (13), and a single bout of exercise reduced the prevalence of hyperglycemia over the subsequent 24-h period (26). A systematic review showed that the association between the timing of exercise and the timing of meals plays an important role in improving glucose control in individuals with type 2 diabetes, with more important benefits whether the exercise was conducted between 30 and 60 min after the meal consumption as compared to a no exercise control condition (32).

Considering the hormonal response, insulin levels were increased at 15 minutes and 30 minutes during the exercise but decreased 60 minutes after exercise. Unfortunately, the lack of a resting control trial does not allow us to isolate the effects of breakfast from those of physical exercise. In accordance with observed post-exercise response, a previous study that evaluated aerobic exercise performed at 80% and 120% of the lactate threshold showed decreased insulin levels at the 15th and 45th minute of post-exercise recovery compared to control session (33). In our work, six participants had more than 10 years of disease, and despite they HbA1c is similarly high, the metformin doses and the fasting insulin present wide variation, suggesting that beta-cell status is probably diverse among them, which may have influenced exercise response. Beta-cell function is an important indicator of disease status, and progression of T2D is related to continuous loss of beta-cell function, with higher HbA1c levels and longer duration of T2D being associated with a declining beta-cell function. Although pancreatic functional reserve decreased with the duration of diabetes, beta-cell loss appears to be irreversible in individuals with T2D duration of longer than 10 years. Moreover, the risk of beta-cell stress is about 3.8 times higher for each 1% increase in HbA1c (34).

We found that GLP-1 levels increased at 30 minutes during the exercise. In accordance, one study found higher GLP-1 levels at 1-h post-exercise but not in the control session in obese sedentary males (35), and endurance-trained males showed increased GLP-1 levels immediately after the exercise session (36). However, in type 2 diabetes, no increase in GLP-1 levels was observed after 40 minutes of combined aerobic plus resistance exercise (37). The absence of glucagon changes was unexpected since increased glucagon levels were observed after exercise in previous studies (16,17). Differences in experimental design are a possible explanation of the disagreement between the results of the present study, since in our study, patients performed the exercise session immediately after breakfast, and the previous results were obtained in the fasting state. Increase in glucagon levels after a single bout of exercise was previously reported in diabetic subjects with chronic hyperglycemia, suggesting that the hyperglycemic state influences the hormonal responses to exercise (38).

One limitation of the present study is its small sample size, which impedes deepening the analyses. Second, the subjects with diabetes did not have the best metabolic control, although it was reasonable, so that any extrapolation of our findings should be circumspect. Moreover, beta-cell status is probably diverse, which may have influenced exercise response. In addition, our work is a quasi-experimental study and cannot provide the same information as that from a randomized clinical trial, since we did not test a resting control to isolate the effects of breakfast from those of physical exercise.

In conclusion, subjects with type 2 diabetes presented expected changes in insulin, glucagon and GLP-1 levels after breakfast and a single aerobic exercise session, not accompanied by glycemic variability changes.
